# Delays in Diagnosis and Treatment of Breast Cancer and the Pathways of Care: A Mixed Methods Study from a Tertiary Cancer Centre in North East India

**DOI:** 10.31557/APJCP.2019.20.12.3711

**Published:** 2019

**Authors:** Arvind Kumar, Srabana Misra Bhagabaty, Jaya Prasad Tripathy, Kalaiselvi Selvaraj, Joydeep Purkayastha, Ravikant Singh

**Affiliations:** 1 *Public Health, Cluster Coordinator, Doctors For You, *; 2 *Department of Preventive Oncology, Dr. B. Borooah Cancer Institute, Guwahati, *; 3 *Department of Community Medicine, Assistant Professor, All India Institute of Medical Sciences, Nagpur, India. *

**Keywords:** Breast cancer, delay, mixed method, north east India

## Abstract

**Introduction:**

In India, mortality rate in breast cancer is high because more than half are diagnosed late at locally advanced or metastatic stages. This might be due to presentation delay (recognition of symptoms to first provider consultation) and treatment delay (first provider consultation to initiation of treatment), together known as overall delay. We aimed to estimate the overall delay in diagnosis and treatment in breast cancer and the associated factors, describe pathway of care and explore the reasons for delay from a patients’ and providers’ perspective.

**Methods:**

Explanatory sequential mixed-methods study with a quantitative component (retrospective cohort study including breast cancer patients registered at Dr. Borooah Cancer Institute (BBCI), Guwahati during February-June 2019) followed by descriptive qualitative component (in-depth interviews with 15 patients and 10 care providers).

**Results:**

Of 269 breast cancer patients, median (Inter Quartile Range) overall delay was 203 (110-401) days, presentation delay was 35 (10-112) days and treatment delay was 130 (75-258) days. Majority of patients approached private sector (190, 70.6%) as the first care provider. Nearly half of all patients (136, 50.6%) visited one health care provider before reaching the BBCI and another one-third (90, 33.5%) visited two providers. Reasons for presentation delay were misconception about the disease, perceived stigma, fear and denial of cancer, attribution of symptoms to trivial conditions, family responsibilities and embarrassment of breast examination by a male doctor. Treatment delay was due to initial visit to, misclassification of disease severity, dissatisfaction with care at public facilities, poor accessibility and affordability, fear of treatment and its side effects.

**Conclusion:**

Treatment delay was the major contributor to overall delay. Private providers need to be sensitized and trained in screening of breast cancer and referral of suspected cases of cancer. More awareness is needed about warning symptoms of breast cancer and misconceptions regarding the disease.

## Introduction

Breast cancer is the second most common cancer in the world and the most frequent cancer among women with an estimated 2.08 million new cancer cases diagnosed in 2018 (24.2% of all cancers). It also ranks as the fifth cause of cancer related mortality (15% of all cancer deaths)(International Agency for Research on Cancer, 2018).

Breast cancer survival rates vary greatly, with 5-year survival worse in low-middle-income countries such as Brazil (58.4%), India (52%), Algeria (38.8%), and Gambia (12%) compared to the United States (83.9%), Sweden (82.0%), Japan (81.6%), and Australia (80.7%)(Coleman et al., 2008; Sankaranarayanan et al., 2010). The low survival rates in developing countries are explained by high proportion of women presenting with late-stage disease at diagnosis, lack of adequate diagnosis and treatment facilities leading to delays in diagnosis and treatment (Coleman et al., 2008; Sankaranarayanan et al., 2010). Increased delay is associated with more advanced stage cancer at diagnosis, thus resulting in poorer chances of survival (Caplan, 2014). A meta-analysis by Richards and colleagues demonstrated that women with delays longer than 3 months had shorter survival compared to women who started treatment within the first 3 months of symptom recognition (Richardset al., 1999). Several patient level factors such as advanced age, being single, Hispanic or black, and low educational attainment have been linked to delay in treatment seeking in previous studies.(Rivera-Franco and Leon-Rodriguez, 2018) Two systematic reviews of both qualitative and quantitative studies in Africa have reported patient and health system related barriers contributing to late presentation of breast cancer (Donkor et al., 2015; Espinaet al., 2017).

In India, breast cancer has surpassed cervical cancer as the most common cancer among women. (Indian Council of Medical Research, n.d.) Although the incidence of breast cancer in India is lower than that in the Western countries, the mortality rates are disproportionately higher with 87, 090 deaths each year (Leong et al., 2010). This could be due to presentation delay (PD) or treatment delay (TD) (Rivera-Franco and Leon-Rodriguez, 2018). PD corresponds to delay in seeking medical attention after breast cancer symptoms and TD is delay in diagnosis and treatment after seeking medical care.

Few studies from India have focused on the socio-demographic and clinical factors associated with PD (Gangane et al., 2016; Thakur et al., 2015). However, studies qualitatively exploring the reasons for delayed presentation in Indian context are rare. The mean delay from symptom recognition to first consultation (PD) is largely unknown, except for a single study among 80 patients which reported a delay of ten months (Paksereshtet al., 1970).

In India, health care is predominantly sought from the private sector, largely comprising of individual providers, who are both formal (qualified) and informal (non-qualified) providers. The poor and marginalized communities usually seek health services from informal providers and other alternate systems of medicine. Thus, even after seeking health care there is a delay in definitive diagnosis and initiation of treatment (TD). The reasons for this delay are less studied in the literature. A recent systematic review on delayed diagnosis of breast cancer in LMIC had expressed the need for evidence on the factors related to TD, both health system and patient related (Sankaranarayanan et al., 2010). Quantifying these delays and understanding the pathways of care the patients follow will guide appropriate interventions.

In India, the highest incidence and mortality rate of cancer is seen in the north-eastern (NE) part of India.(ICMR - National Centre for Disease Informatics and Research, 2016) Assam is the biggest state in the NE region with difficult geographical terrain, prone to natural disasters like heavy rainfall, floods, earthquakes throughout the year. With limited facilities for cancer care in the region and difficult terrain, access to appropriate health care is a major barrier.

The Dr. Bhubaneswar Borooah Cancer Institute (BBCI) is the only Regional Cancer Centre (RCC) which caters to cancer patients in the entire north-eastern region. In 2017, nearly 750 patients with breast cancer were registered in the hospital, of whom nearly 75% were reported in the later stages (stage III and IV). There is meagre information about the time taken for seeking care, getting diagnosed and treated for breast cancer. We also do not know the exact reasons for these delays which require an in-depth qualitative exploration.

Against this background, we conducted this study among the breast cancer patients registered during February to June 2019 at BBCI in Guwahati, India, to: 

i) Determine the proportion of patients with overall delay (more than 3 months from symptom recognition to treatment initiation) and the socio-demographic and clinical factors associated with it, 

ii) Determine the median time from symptom recognition by the patient and first health care provider (HCP) contact (presentation delay) and from the first HCP contact to beginning of treatment (treatment delay), 

iii) Describe the first health care provider and the pathway of the care sought before reaching the RCC, and 

iv) Explore the reasons for this delay from patient’s and provider’s perspectives.

## Materials and Methods

*Study Design*

Explanatory sequential mixed-methods study with a quantitative component ( hospital based cohort study) followed by a descriptive qualitative component (in-depth interviews with patients and health care providers). 

*Setting*

General setting: This study was carried out in BBCI, which is a regional cancer centre in Guwahati, Assam, one of the eight states in the North East (NE) India. Guwahati is the capital city of the Indian state of Assam and also the largest urban area in NE India, populated by nearly 1 million inhabitants as per 2011 census. (Office of the Registrar General and Census Commissioner India, 2011)

Specific setting: The cancer hospital was operational from 1974. In 1980, it was recognized as a Regional Cancer Centre by the Ministry of Health and Family Welfare, Government of India 

This institute caters to patients from the entire north-eastern region of India. Majority of the patients are referred from the district hospital, medical college or the private sector. It is a 230-bedded cancer hospital with various departments like pathology, radiology, surgical and medical oncology, radiotherapy, nuclear medicine, pain and palliative care and preventive oncology. It charges nominally for various services it offers. Majority of the patients avail these services through the Government of Assam cashless health insurance scheme (Atal Amrit Abhiyan) under which individuals below poverty line (BPL) and those in the low income households across the state are entitled upto Rs 2,00,000 (~2890$). About 12,000 new and 80,000 old cancer patients visit the institute every year. It also has a Population and Hospital Based Cancer Registry under the National Cancer Registry Programme of Indian Council of Medical Research. The hospital is equipped with advanced cancer diagnostic and treatment modalities under the departments of radiotherapy, radio-diagnosis, pathology and surgical oncology. There is a 26 bedded day care chemotherapy ward in addition to indoor beds. Breast cancer services are provided at outpatient and inpatient units. 

*Study participants*

For the quantitative phase, the study participants included all breast cancer patients registered at BBCI during February-June 2019. Newly registered or diagnosed female patients without definite treatment date and male breast cancer patients were excluded. Data collection was done between February-June 2019. 

For the qualitative phase, patients with breast cancer seeking care from BBCI and health care providers at different levels of care within the same district constituted the study participants. The patients who came to the hospital OPD and gave consent for interview were recruited.

*Data variables, sources of data and data collection*

*Quantitative data collection*

The data on socio-demographic and clinical details, medical history, reproductive history, habits and tumor related characteristics were extracted from the patient case sheet. Information on PD and TD (date of recognition of symptom, type and number of health care providers visited, date of visit, date of definitive diagnosis and treatment) were collected using a structured questionnaire which was self-administered. As much as possible, the date of diagnosis and initiation of treatment were extracted from patient clinical records. All breast cancer patients who came to the OPD during the study period were recruited consecutively and interviewed by the Principal Investigator. 

*Source of data was the patient case sheet, structured interview proforma and patient’s previous clinical records.*

*Qualitative data collection*

The reasons for delay in health care seeking were explored through in-depth interviews (IDIs) with breast cancer patients who presented late (i.e. 3 months after symptom recognition). Those who were willing and gave consent for interview were included. A total of 15 interviews were conducted. The final sample size was decided based on saturation of findings. The interviews were conducted in local language (Bengali) or Hindi in which both were familiar with.

HCPs including the Oncologists (n=2), staff nurse (n=1) and medical social worker (n=1) at the BBCI. A total of four medical officers were interviewed at various levels of health care such as the block PHC (in rural areas, n=2), state dispensary (in urban areas n=1) and the medical college (n=1). In addition, three community health officers were also interviewed at the health and wellness centres in rural areas. These health facilities were chosen conveniently within the same district as the BBCI.

The principal investigator (PI), who is a male doctor (Master in Public Health), and trained in qualitative research methods conducted the IDIs after obtaining their consent to participate in the study. The PI was accompanied by a female nurse so that the female participants feel comfortable. The patient interviews were conducted in a separate room adjacent to the OPD in order to ensure patient privacy and confidentiality. Interviews with HCPs were done at their respective health facility. The participants were informed about the purpose of the study prior to the interview. Only the participant and the researcher along with a note taker were present during the interview. A pre-tested topic guide with broad open-ended questions was used to guide the interview. Audio recording (after consent) and/or verbatim notes were taken during the interview. After the interview was over, the summary of the interviews was read back to the participants to ensure participant validation. 

*Analysis and statistics*

*Quantitative*

Quantitative data were double entered and validated using EpiData version 3.1 and analysed using EpiData analysis V2.2.2.182 (EpiData Association, Odense, Denmark) and STATA version 13. 

Socio-demographic and clinical profile of the patients was summarized using numbers and proportions. Delays were summarized using median (interquartile range) number of days. The association between delay (> 3 months following symptom recognition) and socio-demographic and clinical factors was assessed using log binomial regression. A 3 month cut-off was used to define delay because there is convincing evidence in the literature indicating that women with delays longer than 3 months have shorter survival compared to women who started treatment within the first 3 months of symptom discovery. The measure of association has been presented using adjusted Relative Risks (aRRs) with 95% confidence interval. The final regression model included variables like: age group, education, tobacco use, distance of nearest PHC, first healthcare provider, age at first child birth, physical activity, stage of cancer.

*Qualitative*

Following each interview, transcripts were prepared by the PI as soon as the interviews were over based on the audio recordings/notes in English language. Manual descriptive content analysis was used to analyze the transcripts (Creswell and Plano Clark, 2007; Kvale, 2007). The transcripts were read several times by two investigators (AK and KS) and relevant codes were identified related to the global theme of ‘barriers to late presentation’. Similar codes were combined to generate themes using standard procedures and in consensus (Saldana, 2010). Any difference between the two was resolved by discussion or by a third investigator (JPT), whenever required (Creswell and Plano Clark, 2007). The findings have been reported by adhering to the ‘Consolidated Criteria for Reporting Qualitative Research’ (COREQ) guidelines (Tong, Sainsbury, and Craig, 2007).

*Ethical issues*

Ethics Approval: Ethics approval was obtained from the Institutional Ethics Committee, BBCI, Guwahati, Assam, India (BBCI-TMC/334/2019) and the Ethics Advisory Group of the International Union Against Tuberculosis and Lung Disease, Paris, France (100/18). Written informed consent was obtained from the participants before conducting interviews. 

## Results

A total of 469 patients were recruited for the quantitative part of the study. For the qualitative component, 15 patients and 10 HCPs were interviewed.

Majority of the breast cancer patients were aged 45-64 years (162, 60.2%), married (235, 87.4%), stayed in rural locations (195, 72.5%) and were homemakers (230, 85.5%). Nearly one-fourth of them belonged to the lower socio-economic class (74, 27.5%). The median (IQR) distance of the patient homes from the Regional Cancer Centre was 150 (80-255) kilometers. Of the first care providers visited by the patient, private sector (190, 70.6%) was the most common followed by primary health centre (27, 10%) and district hospital (20, 7.4%). Nearly half of all patients (136, 50.6%) visited one health care provider before reaching the BBCI and one-third (90, 33.5%) visited two providers. Table 1

Table 2 shows the clinical and tumor related characteristics of breast cancer patients. The most common symptom was lump (259, 96.3%) followed by pain in breast (88, 32.7%). The histopathological finding of FNAC was poorly differentiated (grade III) in 55.4% (n=149) of cases. Majority of the tumor (167, 62.1%) was 2-5 cm in size. Nearly two-third of the patients (175, 65%) came in advanced stages of cancer (stage 3 and 4). About half of the patients (136, 53.1%) had an overall delay (symptom recognition to initiation of treatment) of more than three months.

Table 3 shows overall delay, presentation delay in care seeking and treatment delay by socio-demographic characteristics. Median (IQR) overall delay was 203 (110-401) days, presentation delay was 35 (10-112) days and treatment delay was 130 (75-258) days. No significant association was seen between socio-demographic characteristics and delays except level of education. There was a significant downward trend in delay with increasing level of education.

Table 4 describes the socio-demographic, behavioral and clinical characteristics associated with overall delay. Current users of tobacco (aRR=1.6, 95% CI: 1.1-2.2) were more likely to be associated with delay whereas elderly patients (aRR=0.5, 95% CI: 0.2-1.0) were less likely to delay more than 3 months.

**Figure 1:**
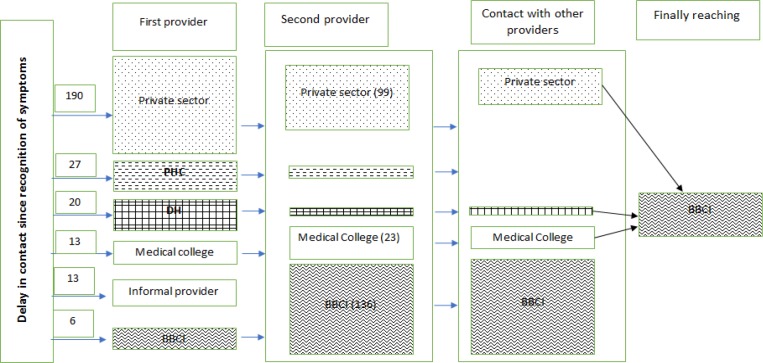
Pathways1 of Care among Breast Cancer Patients Registered at Dr Bhubaneswar Borooah Cancer Institute, Guwahati, India During February-June 2019. BBCI, Dr B Borooah Cancer Institute; DH, District Hospital; PHC, Primary Health Centre

Figure 1 shows the pathway of care and HCPs visited by the patients before reaching the BBCI. Majority of the patients sought consultation from the private sector followed by district hospital and medical college before reaching the cancer care centre. Some patients also approached informal providers as their first provider consultation.

A total of 15 patients (age range: 35-65 years; 70% in advanced stage of disease) and 10 HCPs were interviewed. Three major themes emerged around the global theme of ‘Reasons for delay in seeking care and treatment for breast cancer’: i) reasons for presentation delay, ii) reasons for treatment delay, and iii) suggestions to reduce the delay

*i) Reasons for presentation delay*

*Misconceptions and beliefs regarding the disease*

Misconception and wrong belief about breast cancer were expressed by some of the women in the study. Causes of breast cancer were strongly attributed to evil spirits, immoral lifestyle choices, and wrong deeds in the past. It is believed to be an incurable disease.

*“In television we watch movie where people with cancer always die. This is the common perception that I grew up with” (patient, 46 years female).*

*“The general belief in Assam is that if you have cancer why are you wasting money in the treatment since anyway you are going to die”“Some think that the disease is due to some bad karma (deeds)in the past” (patient, 51 years female).*

It appeared that women’s negative view about breast cancer was uniform and stigmatizing, prolonging presentation. Another major reason that appeared that women delayed seeking care due to Fear of being diagnosed with cancer (PHC Medical officer, 26 years old Male Provider MBBS)

*Stigma related to the disease*

Stigma due to cancer diagnosis was described as a barrier to screening, early diagnosis and treatment seeking for women with symptoms. Cancer stigma emerged as a general theme across transcripts as descriptions of how women with breast cancer would be treated and looked upon by husbands, family and the community. 

A medical officer narrated,“*Family members feel shy to go for diagnosis and treatment, they don’t come forward due to the fear of being outcast by the society” (PHC medical officer MBBS)*

*“Many people don’t want to marry a girl if she has breast cancer history in her family” (PHC medical officer, 29 yrs MBBS MD)*

*“she feels embarrassed about the disease. Maybe she is shy to come for screening thinking what others in the society or her husband will think about her if she goes for breast cancer screening or examination”(PHC medical officer MBBS)*

**Table 1:** Socio- Demographic and Behavioral Characteristics of Breast Cancer Patients Registered at Dr Bhubaneswar Borooah Cancer Institute, Guwahati During February-June 2019 (N=269)

Characteristics	N			(%)	
Age group					
25-44 years	90			33.5	
45-64 years	162			60.2	
65 years and above	17			6.3	
Marital status					
Married	235			87.4	
Unmarried	16			5.9	
Widow	18			6.7	
Education					
No Formal Education	70			26	
Primary	34			12.6	
Middle	43			16	
Secondary	59			22	
Higher Secondary	36			13.4	
Graduate and above	26			9.6	
Not recorded	1			0.4	
Occupation					
Home maker	230			85.5	
Daily wage	5			1.9	
Farmer	1			0.4	
Salaried	16			5.9	
Business	5			1.9	
Others	9			3.4	
Not recorded	3			1.1	
Residence					
Rural	195			72.5	
Urban	73			27.1	
Not recorded	1			0.4	
Religion					
Hindu	204			75.8	
Muslim	48			17.8	
Others	17			6.3	
Socio-economic status*					
Lower class	74			27.5	
Middle class	67			24.9	
Upper class	128			47.6	
Smoking					
Never smoked	260			96.7	
Past smoker	6			2.2	
Current smoker	2			0.7	
Not recorded	1			0.4	
Smokeless tobacco use					
Never	170			63.2	
Past	54			20.1	
Current user	44			16	
Not recorded	1			0.4	
Characteristics	N			(%)	
Distance from RCC(median/IQR)a	150			80-255	
Distance of nearest PHC (median/IQR) a	6			3-10	
Age at marriage (median/IQR)	20			(17-25)	
Age at birth of first child (median/IQR)	22			(19-26)	
First health care provider					
Private sector	190			70.6	
Primary health centre	27			10	
District hospital	20			7.4	
Medical College	13			4.8	
Informal provider	13			4.8	
BBCI#	6			2.2	
HCPs visited before reaching BBCI				
0		6	2.2	
1		136	50.6	
2		90	33.5	
3		27	10	
4		9	3.3	
5		1	0.4	

#BBCI, Dr. B Borooah Cancer Institute; HCP, Health Care Provider

**Table 2:** Tumor Related Characteristics of Breast Cancer Patients Registered at Dr Bhubaneswar Borooah Cancer Institute, Guwahati, India during February –June 2019

Characteristics	N	(%)
FNAC done		
BBCI	182	(67.7)
Public	68	(25.3)
Private	6	(2.2)
Not recorded	13	(4.8)
Histological Grade		
Grade I: well differentiated	11	(4.1)
Grade II: moderately differentiated	109	(40.5)
Grade III: poorly differentiated	149	(55.4)
Tumor size		
≤2 cm	23	(8.6)
2-5 cm	167	(62.1)
>5 cm	53	(19.7)
>5 cm and chest wall and skin	26	(9.7)
Clinical staging		
Stage 1	14	(5.2)
Stage 2	80	(29.7)
Stage 3	147	(54.6)
Stage 4	28	(10.4)
Category of delay*		
Overall delay (more than 3 months)	136	(53.1)
No delay (less than 3 months)	120	(46.9)
Clinical symptoms		
Lump or swelling	259	(96.3)
Pain in breast	88	(32.7)
Change in skin texture	09	(3.3)
Change in color	10	(3.7)
Nipple retraction	7	(2.6)
Nipple discharge	12	(4.5)

*Delay from symptom recognition to treatment initiation; BBCI, Dr Bhubaneswar Borooah Cancer Institute

**Table 3:** Delay in Seeking Care, Diagnosis and Treatment among Breast Cancer Patients Registered at Dr Bhubaneswar Borooah Cancer Institute, Guwahati, India during February-June 2019

Characteristics	Total	Overall delay	p-value†	Presentation delay	p-value	Treatment delay	p-value
Age group (in years)			0.3		0.8		0.8
25-44	86	204 (107-407)		43 (8-144)		114 (75-260)	
45-64	150	225 (119-415)		31 (10-88)		148 (76-272)	
65 and above	17	151 (102-336)		60 (12-140)		91 (74-196)	
Education status			0.003		0.01		0.03
No formal education	66	239 (125-493)		61 (15-181)		136 (75-236)	
Up to primary education	32	226 (122-527)		34 (18-106)		203 (82-460)	
Up to secondary	96	193 (118-335)		33 (10-101)		139 (76-268)	
Higher secondary and above	59	149 (93-337)		31 (7-71)		100 (68-241)	
Socio-economic status			0.9		0.6		0.9
Upper class	74	235 (107-403)		40 (10-159)		128 (74-271)	
Middle class	67	160 (110-276)		30 (5-61)		116 (70-221)	
Lower class	128	238 (123-415)		33 (13-87)		163 (85-269)	
Place of residence			0.3		0.23		0.8
Rural	182	187 (118-401)		33 (8-88)		133 (80-256)	
Urban	71	232 (102-441)		36 (10-174)		128 (66-276)	
Marital status			0.6		0.001		0.4
Married	223	203 (117-415)		35 (8-95)		131 (76-265)	
Unmarried	16	153 (95-397)		18 (8-97)		92 (71-392)	
Widow	14	242 (131-308)		78 (31-140)		150 (64-227)	
Overall delay	253	203 (110-401)		35 (10-112)		130 (75-258)	

Figures expressed in the table represent number of days of delay in the form of median (interquartile range); †Kruskal-Wallis ANOVA, two-sided, Pvalue<0.05

**Table 4:** Socio-Demographic and Clinical Factors Associated with Overall Delay among Breast Cancer Patients Registered at Dr Bhubaneswar Borooah Cancer Institute, Guwahati, India During February-June 2019

Characteristics	Total	Increased delay (>3 months)			RR [95% CI]		p value†		aRR [95% CI]		p value	
	N	N		(%)								
Age group												
25-44 years	87	45		51.7	Ref		-		Ref			
45-64 years	152	86		56.6	1.1 (0.9-1.4)		0.5		1.0 (0.8-1.3)		0.98	
65 years and above	17	5		29.4	0.6(0.6-1.2)		0.1		0.5 (0.2-0.96)		0.05	
Education												
No Formal Education	66	39		59.1	Ref				Ref			
Primary	33	20		60.6	1.0 (0.7-1.4)		0.9		1.1 (0.8-1.5)		0.6	
Upto secondary	97	51		52.6	0.9 (0.7-1.2)		0.4		1.0 (0.7-1.3)		0.9	
Higher Secondary and above	60	26		43.3	0.7 (0.5-1.0)		0.08		0.8 (0.6-1.2)		0.3	
Occupation									-			
Home maker	230	118		51.3	Ref							
Manual workers	18	10		55.6	1.1 (0.7-1.7)		0.7					
Office related works	21	8		38.1	0.7 (0.4-1.3)		0.3					
Residence												
Rural	184	94		51.1	Ref				-			
Urban	72	42		58.3	1.1 (0.9-1.5)		0.3					
Religion												
Hindu	204	105		53.6	Ref				-			
Muslim	48	23		52.3	1.0 (0.7-1.3)		0.9					
Others	17	8		50	0.9 (0.6-1.6)		0.8					
Socio-economic status									-			
Upper class	47	26		55.3	0.8 (0.5-1.2)		0.2					
Lower middle class	34	14		41.2	1.0 (0.8-1.4)		0.9					
Lower class	169	91		53.9	Ref							
Tobacco use												
Never user	156	74		47.4	Ref				Ref			
Past user	55	37		67.3	1.4 (1.1-1.8)		0.01		1.2 (0.9-1.5)		0.1	
Current user	43	25		58.1	1.2 (0.9-1.7)		0.2		1.6 (1.1-2.2)		0.01	
Distance from RCC in kms									-			
Within 100 Kms	93	51		56.7	Ref							
100-200	78	40		54.8	1.0 (0.7-1.3)		0.8					
More than 200	98	45		48.4	0.9 (0.6-1.1)		0.3					
Distance of nearest PHC in kms												
Within 5 Kms	122	60		49.2	Ref				Ref			
6-10	78	50		64.1	1.3 (1.0-1.7)		0.03		1.2 (0.9-1.5)		0.2	
More than 10	55	26		47.3	1.0 (0.7-1.3)		0.8		1.0(0.7-1.4)		0.98	
Physical Activity (hours/week)												
0-3	86	54		62.8	Ref				Ref			
4-4	81	45		55.6	0.9 (0.7-1.1)		0.3		0.9 (0.7-1.2)		0.5	
More than 14	87	36		41.4	0.7 (0.5-0.9)		0.01		0.7 (0.5-1.0)		0.03	
Age at First child birth												
Within 20 years	80	52		65	Ref				Ref			
21-25	95	38		40	0.6 (0.5-0.8)		0.001		0.8 (0.6-1.0)		0.08	
25-38	57	38		66.7	1.0 (0.8-1.3)		0.8		1.2 (0.9-1.6)		0.2	
First initiative for treatment seeking												
Self	213	110		51.6	Ref				-			
Health Staff	32	15		46.7	0.9 (0.6-1.3)		0.006					
Family member	11	11		100	-							
Characteristics	Total	Increased delay (>3 months)				RR [95% CI]		p value†		aRR [95% CI]		p value	
	N	N	(%)										
Stage													
I	14	8	57.1			Ref				Ref			
II	73	48	65.8			1.2 (0.7-1.9)		0.6		1.2 (0.7-1.8)		0.5	
III	141	70	49.7			0.9 (0.5-1.4)		0.6		1.0(0.6-1.6)		0.9	
IV	28	10	35.7			0.6 (0.4-0.9)		0.2		0.7 (0.4-1.4)		0.3	

RCC, Regional Cancer Centre; PHC, Primary Health Centre; IQR, Inter Quartile Range; †P Value < 0.05

*Lack of family support*

Women are also reluctant to share their health issues with their family or husband due to lack of family support.

*“married women in our community are not self-dependent, they rely on their husband. So, for each matter they have to ask their husband and, in such cases, husband may not allow her to go out to seek health care” (Medical Officer with Post graduate Qualification)*

*“in laws in our family do not allow their daughter-in-law to get her private parts checked by a doctor”(Medical Officer with Post graduate Qualification)*

*Priority to family and social responsibilities*

Women’s social responsibilities of caring for children, husband and relatives had an impact on their delayed presentation. Due to these obligations, some women postponed going to the hospital for months. 

*“I have 3 children and all the while I was thinking of them. If something happens to me who will take care of them. They are small and they want me all the time. Me and my husband were terrified” (patient, 39 years female)*

*Attributing symptom to benign conditions*

Immediately after detecting abnormal symptoms, women were more likely to perceive their symptom as a benign or trivial condition.

*“I didn’t even think that this might be cancer. I thought it’s kind of a boil and will go away” (patient, 39 years female)*

*Attitude of neglect*

Women tend to neglect their own health due to other perceived priorities within their family and the society or probably due to their casual attitude towards their own health. A doctor from his clinical experience opined, 

*“when we ask them, we get to know that lump has been there for 1- 2 years like that and they have ignored this” (Resident doctor, RCC)*

*Embarrassment of breast examination*

*Many patients and health professionals reported embarrassment to perform breast examination by a male doctor as a barrier to seeking professional help.*

*“More than fear and pain, I think it is embarrassment of letting someone else have a look at their breast or touch it”* (Resident doctor, RCC)

A Medical Officer said, “*The gender of the person who is examining the patient matters a lot. Most women don’t like to be examined by a male docto*r” (Rural Health Practitioner [28yrs])

*Fear and denial of cancer*

Fear of the diagnosis of cancer made some patients hesitant to approach a facility for screening or definitive diagnosis.

*“people have the fear of getting diagnosed with breast cancer before going for a screening”*(Rural Health Practitioner [28yrs])

*“They have the fear of death after being diagnosed with breast cancer” (staff Nurse, 23 years Female)*

*Denial*

*“Cancer never came to anyone’s head because there is always a feeling that cancer happens to someone else and it can’t happen to me”(Patient, 46 years female)*

A patient emotionally said, *“Till the last moment I was sure it can’t be cancer. I just cannot have cancer in just 38 years of life. I have never touched alcohol or cigarette. So why only me? I cried and cursed God for it” (Patient, 42 female)*

*ii) Reasons for treatment delay*

*Affordability and accessibility*

Money and distance to the nearest health facility were other barriers to accessing the health system.

*“everything is so expensive, collecting money took a lot of time” (patient, 42 years female)*

*“Money was a big barrier. My husband worked extra for it. Even during my treatment, he was working then coming to me and then working again” (patient, 38 years female)*

*Alternate therapies*

Due to the pluralistic nature of the Indian health system, women preferred private consultations, other alternate systems of medicine, informal providers and faith healers and over the counter drugs in lieu of modern medicine. So, they visit several providers before reaching the appropriate facility for definitive diagnosis of cancer.

A patient honestly confessed, *“We are a little superstitious people so we approached an informal provider to do jharphoonk (incantation) and left it for 3 years”* (patient, 40 years female)

A doctor said, *“They will take help from some magic gurus and come to hospitals late*” (Resident doctor, RCC)

*Fear of treatment and its side effects*

There is a general perception in the society that treatment of breast cancer, rather any cancer is costly, invasive and painful with lots of side effects which probably explains pre-treatment delay. 

A medical officerwas quoted saying, *“it will cause mental as well as emotional trauma. Thinking of undergoing mastectomy can be emotionally challenging for a female due to disruption of her physical structure” *(Medical Officer, Post graduate qualification)

A patient said, *“My family thinks that if someone gets cancer then people should go out of the state for their treatment. They do not know that it can be treated here also. If we go outside for treatment it will be costly” *(Patient, 48 years female)

A patient recollected thought prior to her treatment, *“I didn’t lose my hair which I was very scared of at the beginning of the treatment. I had no other idea about what is going to happen to me during or after the treatment” *(Patient, 36 years female)

*Dissatisfaction with the health care service delivery*

Patients reported dissatisfaction with the quality of service, attitude of the doctors and nurses and long waiting queues at public health facilities. These bitter experiences force patients to consult the private sector or forego treatment if somebody is already on treatment.

A patient during her treatment shared her experience, *“people over there are always in a hurry, so they hardly listen to me including the doctors. There are so many instances whether doctor and even the nurse have replied rudely when we ask something. It is very uncomfortable to repeat a questionalso”* (Patient 32 years female)

Another patient said, *“I do not like their way of dealing with the patients. Their tone becomes stronger when you ask them anything the second time. Manytimes, I used to see the junior doctors only, I never get satisfied in my mind though they check my report properly” *(Patient,52 years female)

*Delay in diagnosis of breast cancer*

Patients have experienced delay in definitive diagnosis due to the inability of the doctor in suspecting cancer. She says *“Surprisingly in a big town like Sibsagar the doctor couldn’t find out that this can be cancerous. She also gave me the same medicine for a month” *(Patient, 39 years female)

*Poor performance status / debilitating nature of the patient*

The treating surgeon had opined that “when the diagnosis is confirmed, for further management the discussion happens in the tumour board (multi-disciplinary team). During the review, if the patient is not fit enough to undergo surgery due to poor performance status or poor control of comorbid conditions (Karnofsky performance status) they will not be initiated on treatment at once. *“If there is lot number of patients there could be long waiting list for surgery”*

*iii) Suggestions to reduce delay*

Some of the suggestions to reduce delay from the providers’ perspective are: i) free decentralized screening facilities, ii) improve awareness about breast cancer symptoms, screening, diagnosis and treatment through multiple channels of communication, iii) allay fears, bust myths and misconceptions about the disease, iv) promote breast self-examination through female health workers, v) opportunistic screening at health facilities, vi) patient friendly gender sensitive screening, as a doctor said, “*We need to make the screening site patient friendly, taking into account gender issues. If the staff are not patient friendly, if they misbehave and if they don’t make the patient feel comfortable then obviously it will affect in the uptake of breast cancer screening*” vii) enhanced role of women support groups where they can openly discuss about such sensitive issues.

## Discussion

This mixed methods study highlights the presentation and treatment delay in seeking care, diagnosis and management of breast cancer and the reasons for this delay. There were some interesting findings in the study which are discussed below.

Patient level delay was found to be around one month in this study which is much lower than other studies from India and other Asian countries where PD ranged from 6-8 months with more than half reporting after 3 months of experiencing symptoms (Gangane et al., 2016; Pakseresht et al., 1970; Thakur et al., 2015). In Sub-Saharan African nations, presentation delay was even higher around 12-13 months (Galukande, 2014; Odongo, Makumbi, Kalungi, and Galukande, 2015). This is in contrast to studies done in developed countries where median delay to the first consultation was 9-61 days (Rivera-Franco and Leon-Rodriguez, 2018).

Treatment delay (130 days) was found to be much more than patient level delay (35 days). Although it is encouraging to note that patients seek consultation early, it reflects poorly on the health system. There is scanty literature on this delay in India. A prospective cohort from India by Chintamani et al. also had similar findings with delay at the provider end being significant mostly due to unregistered doctors or quacks (Chintamani et al., 2011). In this study, majority of the patients sought care from multiple private providers. It seems as if they are unable to suspect the disease and refer cases timely for appropriate management.

This study found no association of key demographic variables with overall delay. Demographic factors have little role to play in influencing delay. Previous studies have also highlighted the fact that psychosocial and cultural factors were the main predictors of delay rather than socio-demographic characteristics (Nosarti et al., 2000). A systematic review also found contradictory results about the association of socio-demographic factors such as age, education, marital status, occupational status and socio-economic status (Rivera-Franco and Leon-Rodriguez, 2018). This could also mean that awareness and efforts to reduce the psychosocial aspects need to be directed towards all economic classes equally. 

The present study showed that women tend to attribute their symptoms (especially painless lumps) to trivial conditions, thus contributing to delay in accessing health care. Similar findings have been reported in other studies (Khakbazan et al., 2014; Nosarti et al., 2000). A study by Nosarti et al., (2000) on 692 patients showed that women attributed breast symptom to less serious conditions, which was reported as the most common reason for delayed presentation.

Social obligations, family responsibilities and burden of work were common barriers that influenced decision to seek care. It is well known that women prioritize other matters and neglect their own health (Bonsu and Ncama, 2019; Majaj et al., 2013).This is similar in case of women with breast cancer as well where parenting and other family and social issues influence their health seeking behaviour (Bonsu and Ncama, 2019; Moodleyet al., 2016; Vanderpool et al., 2013).

Women generally prefer private providers, informal providers, religious leaders or practitioners of traditional medicine probably due to misconceptions and beliefs about the disease or lack of trust in the government system or social stigma attached to the disease. Some even resort to self-medication from nearby pharmacies for temporary symptomatic relief. This leads to a delay in seeking appropriate care. Use of traditional medicine, self-medication and religious leaders has been reported in cancer studies across African and Indian communities (Bonsu and Ncama, 2019; De Ver Dye et al., 2011; Ukwenya et al., 2008).

Perceived stigma associated with breast cancer within the Indian society emerged in this study. This results in non-disclosure of symptoms even within the family as they feel shy enough to discuss it. Lack of support from the family members including her husband and in-laws contributes to this behaviour.Further research on cancer stigma is needed to provide insight for future programmatic efforts to reduce cancer stigma.

Accessibility was a major barrier to seeking appropriate care as BBCI is the only specialised cancer care centre in the NE Region with difficult terrain and landscape. The patients had to visit the facility repeatedly for a battery of tests before getting diagnosed. With the median distance from their homes to the BBCI being 150 kilometres (from the quantitative findings), it is certainly going to be a tough ask for them. Therefore, they seek care from private providers, other alternate providers or public health facilities which are located nearby before finally landing at BBCI after a long delay. 

Though to prevent the delay, the health system is improving the access to care through decentralised definitive care at district hospitals and reimbursement of incurred expenditure from health insurance schemes often patient’s performance status (fitness to undergo surgery) does not let the surgery or radiotherapy to be initiated at the earliest. Since in this study around two third of patients are diagnosed with late stage of disease and often the late stage is associated with poor performance status this could be one of the major limiting factors for timely initiation of definitive treatment. 

The study had three strengths. First, a mixed methods approach enabled an understanding of the reasons for delay in seeking care and also come up with feasible solutions to mitigate the barriers from patients as well as providers across three tier health system. Second, we adhered to the STrengthening the Reporting of OBservational Studies in Epidemiology and COREQ guidelines to report the study findings.(Tong et al., 2007; Von Elm et al., 2014) Third, double data entry and validation is likely to minimise data entry errors. 

There were few limitations in this study. First, patients may have had challenges in recalling information related to their first experience of symptom recognition and the health care providers visited. However, primarily the dates related to diagnosis and treatment were corroborated from available clinical records. Second, social desirability bias could creep in as the patients might respond favourably to questions related to their delay in first presentation and the first health care provider visited. Third, the study participants might be hesitant to respond to a male interviewer (PI), however, a female nurse also accompanied the PI during the interviews. Fourth, this study only included those patients who reached the BBCI to receive cancer care excluding those who died or those who did not receive appropriate care for breast cancer. Fifth, in this study we did not collect the patient’s performance status at the time of diagnosis. During the qualitative interviews patient performance status was emerged as one of the factors treatment delay.

The study results have some programmatic implications. First, more awareness needs to be raised about warning symptoms of breast cancer, importance of early diagnosis and management of the disease. Misconceptions regarding the disease need to be tackled through culture sensitive messaging via multiple channels of communication. Second, since majority of the patients initially approach the private sector, private providers need to be sensitized and trained in screening of breast cancer and referral of suspected cases of cancer. Public Private Partnership models could be harnessed to reduce delay in presentation, diagnosis and treatment of cancers including breast cancer. This involves empanelling private providers to offer diagnosis and treatment of cancers at subsidised rates to make cancer care accessible and affordable. Third, health system delay should be tackled through interventions at different levels of care in terms of trained manpower and facilities for diagnosis and management.
